# The role of immune checkpoints in cardiovascular disease

**DOI:** 10.3389/fphar.2022.989431

**Published:** 2022-10-03

**Authors:** Laura I. Yousif, Anniek A. Tanja, Rudolf A. de Boer, Arco J. Teske, Wouter C. Meijers

**Affiliations:** ^1^ Department of Experimental Cardiology, University Medical Center Groningen, Groningen, Netherlands; ^2^ Graduate School of Life Science, Utrecht University, Utrecht, Netherlands; ^3^ Department of Cardiology, Thorax Center, Erasmus University Medical Center, Rotterdam, Netherlands; ^4^ Department of Cardiology, University Medical Center Utrecht, Utrecht, Netherlands

**Keywords:** immune checkpoint inhibitors, immune checkpoints, myocarditis, cardiomyopathy, atherosclerosis, PD-1, CTLA-4, LAG-3

## Abstract

Immune checkpoint inhibitors (ICI) are monoclonal antibodies which bind to immune checkpoints (IC) and their ligands to prevent inhibition of T-cell activation by tumor cells. Currently, multiple ICI are approved targeting Cytotoxic T-lymphocyte antigen 4 (CTLA-4), Programmed Death Protein 1 (PD-1) and its ligand PD-L1, and Lymphocyte-activation gene 3 (LAG-3). This therapy has provided potent anti-tumor effects and improved prognosis for many cancer patients. However, due to systemic effects, patients can develop immune related adverse events (irAE), including possible life threatening cardiovascular irAE, like atherosclerosis, myocarditis and cardiomyopathy. Inhibition of vascular IC is associated with increased atherosclerotic burden and plaque instability. IC protect against atherosclerosis by inhibiting T-cell activity and cytokine production, promoting regulatory T-cell differentiation and inducing T-cell exhaustion. In addition, PD-L1 on endothelial cells might promote plaque stability by reducing apoptosis and increasing expression of tight junction molecules. In the heart, IC downregulate the immune response to protect against cardiac injury by reducing T-cell activity and migration. Here, inhibition of IC could induce life-threatening T-cell-mediated-myocarditis. One proposed purpose behind lymphocyte infiltration is reaction to cardiac antigens, caused by decreased self-tolerance, and thereby increased autoimmunity because of IC inhibition. In addition, there are several reports of ICI-mediated cardiomyopathy with immunoglobulin G expression on cardiomyocytes, indicating an autoimmune response. IC are mostly known due to their cardiotoxicity. However, t his review compiles current knowledge on mechanisms behind IC function in cardiovascular disease with the aim of providing an overview of possible therapeutic targets in prevention or treatment of cardiovascular irAEs.

## Introduction

Cancer therapy has taken tremendous strides over the course of 2 decades by targeting immune checkpoints (IC). These T-cell surface membrane receptors provide the secondary signal required to either activate or inhibit the T-cell. As tumor cells harness the ability to express the corresponding inhibitory ligands, they can bind to IC and effectively inhibit T-cell activation, thereby circumventing a potential anti-tumor immune response (Sharma and Allison, 2015). Monoclonal antibodies called immune checkpoints inhibitors (ICI) were developed to prevent this interaction. ICI bind to IC on T-cells or their corresponding ligands on tumor cells, block the inhibitory effect of T-cell-tumor cell interaction and allow opportunity for T-cell activation. Currently there are ICI targeting Programmed Cell Death Protein-1 (PD-1) and its ligand PD-L1, Cytotoxic T-Lymphocyte-Associated Protein 4 (CTLA-4) and Lymphocyte Activation Gene-3 (LAG-3), all of which have shown remarkable and durable potency in various types of tumors (Larkin et al., 2015; Wallin et al., 2016; Overman et al., 2018; Lipson et al., 2021).

Despite tremendous success in cancer therapy, one of the major drawbacks of ICI are immune related adverse effects (irAE). As IC inhibition of T-cells is systemic and not localized to the tumor and its environment, it can cause auto-immune damage to multiple organs (Okazaki et al., 2003; Blansfield et al., 2005; Iwama et al., 2014). A meta-analysis showed an incidence of cardiovascular events of 8.32% (95% CI = 6.35–10.53%) among over 21,000 patients receiving ICI in randomized clinical and while that cardiovascular irAE pose a serious threat to patient health, ranging in severity from mild arrhythmias to myocarditis, the latter with a mortality of up to 50% trials (Salem et al., 2018; Xavier et al., 2022) (Xavier et al., 2022) (Salem et al., 2018). This was prudently observed in another meta-analysis which found that while cardiovascular irAE were among the three least occurring ones (8%), they were the second most leading cause of death (25%) (Wang et al., 2018). Upon diagnosis of cardiovascular irAE, current guidelines indicate temporary or permanent cessation of ICI therapy and immediate treatment with immunosuppressives (Andres and Ramalingam, 2019). Unfortunately, not all patients respond to immunosuppressive and in some cases this method of treatment even increases the chance of death (Tison et al., 2019; Cautela et al., 2020).

There is a need for knowledge on the mechanisms of IC in the cardiovascular system to improve treatment with the otherwise very potent ICI therapy. Once understood, these mechanisms could also provide insight into which patients are more susceptible to developing these life-threatening cardiovascular irAEs, and provide potential therapeutic targets. In this review, we summarize current knowledge on the mechanisms of PD-1, CTLA-4, and LAG-3 in the cardiovascular system and their role in cardiovascular disease (CVD). In addition, we aim to provide an overview of possible therapeutic targets in prevention or treatment of cardiovascular irAEs.

## Immune checkpoints

Immune checkpoint receptors such as PD-1, CTLA-4, and LAG-3 are expressed on the surface of T-cells where they prevent T-cell activation (Sharma and Allison, 2015). T-cell activation occurs due binding of the T-cell receptor (TCR) to major histocompatibility complex I or II (MCH-I/II) and additional co-stimulation through CD28-CD80/CD86 binding, which results in recruitment of multiple molecules, including phosphoinositide 3-kinase (PI3K), to the intracellular part of CD28 (Chen and Flies, 2013). PI3K recruitment activates the PI3K/Akt pathway, which promotes proliferation, differentiation and anti-apoptotic signaling in T-cells (Herrero-Sánchez et al., 2016). The activation of T-cells results in differentiation of CD8 cells into cytotoxic T-cells and CD4 cells into stimulatory T helper cells (T_h_) or inhibitory regulatory T-cells (T_reg_), depending on the cytokines in the environment (Vuong et al., 2022). On the other hand, inhibitory IC can prevent overactivation of the immune system and promote self-tolerance.

PD-1 is expressed on the surface of T-cells and interacts with two ligands, PD-L1 and PD-L2 (Ghiotto et al., 2010). PD-L2 is mainly expressed on macrophages and DCs, whereas PD-L1 is present on hematopoietic cells and tissue cells in various organs (Vuong et al., 2022). Binding of PD-1 to either of its ligands leads to downregulated T-cell activity through downstream SHP-2 signaling and subsequent dephosphorylation and inhibition of the downstream PI3K-Akt pathway, resulting in decreased inflammatory cytokine production, cell survival signals and proliferation (Parry et al., 2005; Gato-Cañas et al., 2017; Willsmore et al., 2021). It is suggested that PD-1 suppression of T-cells normally takes place at later stages of an immune response, in peripheral tissue (Buchbinder and Desai, 2016).

CTLA-4 is located intracellularly and translocated to the surface upon T-cell activation (Parry et al., 2005; Rudd et al., 2009). It binds to CD80 and CD86, with higher affinity than CD28, and suppresses T-cell activation through PI3K downstream signaling inhibition, similar to PD-1 inhibition (Parry et al., 2005; Ronen et al., 2022). Additionally, CTLA-4 can interact with PP2A, which dephosphorylates AKT, quenching the pathway further (Willsmore et al., 2021). This reduces cytokine production in CD8 T-cells and promotes differentiation of CD4 T-cells towards T_reg_ cells (Chen et al., 2009; Zhu et al., 2011). In contrast to PD-1, it is proposed that CTLA-4 suppressed T-cell activation earlier on in an immune response (Buchbinder and Desai, 2016).

LAG-3 is expressed on the surface of activated T-cells and constitutively on T_reg_ cells (Zhang et al., 2017). It is involved in suppressing T-cell expansion, increasing cell death and in T_reg_ function. The receptor is homologous to CD4 and can bind MHC-II with higher affinity (Chen and Flies, 2013; Zhang et al., 2017). Besides MHC-II, additional ligands for LAG-3 include liver sinusoidal endothelial cell lectin (LSECtin), Galectin-3 (Gal-3), and fibrinogen-like protein 1 (FGL1) (Xu et al., 2014; Kouo et al., 2015; Wang et al., 2019). Its intracellular signaling mechanisms remain largely unknown but suggested is an association with and inhibition of the TCR/CD3 activating pathway, resulting in reduced T-cell expansion, and inhibited cytotoxic activity of CD8 cells (Anderson et al., 2016).

## Vascular immune checkpoints

### Atherosclerosis

Recently, studies have linked ICI treatment to an increased risk of myocardial infarction and stroke (Drobni et al., 2020; Oren et al., 2020). In a single-center, matched cohort study, patients receiving ICI had a three-fold higher risk of cardiovascular events, likely through accelerated progression of atherosclerosis (Drobni et al., 2020). Therefore, IC inhibition of T cells is thought to contribute to protection against atherosclerosis, although long-term studies are still lacking.

### Programmed cell death protein 1 in atherosclerosis

**FIGURE 1:**
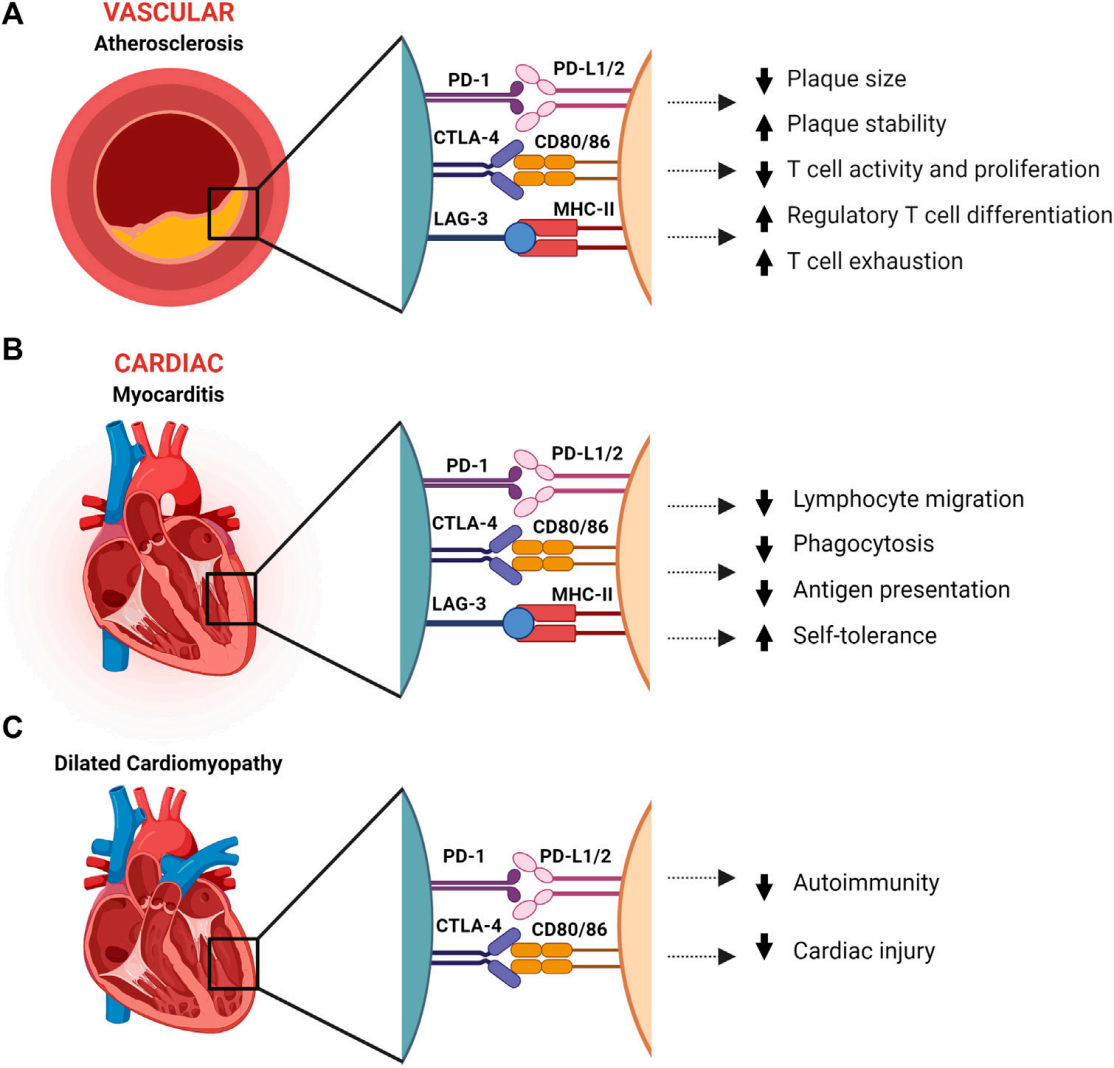
The effect immune checkpoints PD-1, CTLA-4 and LAG-3 in protection against atherosclerosis, dilated cardiomyopathy, and myocarditis. **(A)**. Immune checkpoints have a protective role in atherosclerosis by reducing inflammation and increasing plaque stability. **(B)**. Myocarditis is dampened by IC through reduction of inflammation and T-cell migration, and suppression of autoimmunity. **(C)**. In dilated cardiomyopathy, PD-1 and CTLA-4 are associated with suppressed autoimmunity and reduced cardiac injury. CTLA-4, Cytotoxic T-Lymphocyte-Associated Protein four; LSECtin, liver sinusoidal endothelial cell lectin; LAG-3, Lymphocyte Activation Gene-3; MHC-II, major histocompatibility complex II; PD-1, programmed death-1; PD-L1/2, programmed death-ligand 1/2. Created with BioRender.com.

Protection against atherosclerosis by PD-1/PD-L1 is reflected in knockout mice presenting with enlarged plaques containing higher T-cell and macrophage numbers, increased Tumor Necrosis Factor alpha (TNFα) levels and T-cell activation by antigen presenting cells (APCs), and enhanced cytotoxic activity of CD8 T-cells, all of which increase inflammation and plaque formation (Figure 1A) (Gotsman et al., 2007; Bu et al., 2011). In addition, PD-1 binding to PD-L1-induced differentiated T_reg_ cells inhibits cytokine production of T_h_1 cells, including interferon γ (IFNγ) and TNFα (Frostegård et al., 1999; Vuong et al., 2022). As IFNγ has been identified as a key player in atherogenesis by inducing T-cell and macrophage recruitment, cytokine secretion, and enhanced antigen presentation by endothelial cells, this binding reduces both plaque size and inflammatory T cell responses as shown in Figure 1A (Amento et al., 1991; Gotsman and Lichtman, 2007). IFNγ also contributes to plaque instability by inhibiting vascular smooth muscle cell proliferation and reducing collagen synthesis. Contrarily, T_reg_ cells reduce atherogenisis by secreting anti-inflammatory cytokines, IL-10 and TGF-β, and expressing multiple inhibitory IC, thereby suppressing the proliferation of pro-inflammatory effector T-cells (Saigusa et al., 2020). This is underlined by the observation in mice that depletion of T_reg_, IL-10 deficiency or TGF-β disruption worsens atherosclerotic disease (Robertson et al., 2003; Ait-Oufella et al., 2006). Moreover, when T-cells are continuously exposed to antigen or inflammatory signals, as with TGF-β and IFNy in atherosclerotic lesions, they can become exhausted and lose parts of their effector functions (Figure 1A). This entails reduced T-cell proliferation and cytokine production, and increased inhibitory IC expression, such as PD-1 and LAG-3 (Wherry and Kurachi, 2015). PD-1 expressing exhausted T-cells have been found in atherosclerotic lesions, raising the possibility that by inhibiting PD-1 with ICI, exhausted T-cells are reactivated and contribute to acceleration and exacerbation of atherosclerosis (Fernandez et al., 2019).

On a cellular level, PD-L1 expression on endothelial cells can be induced by IFNγ and TNFα (Mazanet and Hughes, 2022). A study on human umbilical cord vein endothelial cells (HUVECs) demonstrates constitutive expression of PD-L2 and induced expression of PD-L1 after IFNγ treatment (Chen et al., 2016). Oxidized low-density lipoprotein (ox-LDL) impaired HUVECs expressing PD-L1 were able to upregulate CTLA-4 and PD-1 expression on T_reg_ cells and modulated their production of IL-10 and TGF-β. When treated with anti-PD-L1, HUVECs lost their ability to upregulate the IC expression and cytokine production. In addition to trans binding to PD-1 on T-cells, PD-L1 could bind *in cis* to PD-1 on vascular endothelial cells (VECs). This leads to reduced PD-L1 surface expression resulting in enhanced CD8 T-cell toxicity, which causes VECs injury and apoptosis by perforin, TNFα and IFNγ (Veluswamy et al., 2020). Concordantly, endothelial PD-L1/2 blockade enhances IFNγ secretion and lytic activity of CD8 T-cells (Rodig et al., 2003).

It is known from cancer cells expressing PD-L1 that the intrinsic pathway interferes with IFNγ cytotoxicity by inhibiting the downstream JAK/STAT3/caspase7-dependent pathway, thereby protecting against IFNγ-induced apoptosis (Azuma et al., 2008; Gato-Cañas et al., 2017). A similar function of PD-L1 is described in a study in lymphatic endothelial cells that shows protection against apoptosis in lymph node contraction when expressing high levels of PD-L1 (Lucas et al., 2018). All in all, PD-L1/2 expression on endothelial cells demonstrated the ability to inhibit the immune system by upregulating T_reg_ activity and downregulating CD8 T-cell activity, thereby protecting the endothelium against the pro-atherosclerotic effects of immune damage.

### Cytotoxic T-lymphocyte antigen 4 in atherosclerosis

Comparable to PD-1/PD-L1, CTLA-4 knockout mice show increased lesion size, and mice receiving anti-CTLA-4 blocking antibodies showed increased progression of atherosclerosis mainly driven by T-cell-induced inflammation (Figure 1A) (Poels et al., 2020). Corresponding to CTLA-4 knockout studies, mice overexpressing CTLA-4 or receiving CTLA-4 analog abatacept show decreased intimal thickening (58.5% reduction), reduced CD4 T-cell numbers, less proliferation activity and proinflammatory cytokine production (Ewing et al., 2013; Matsumoto et al., 2016). With regards to cellular expressions of CTLA-4 ligands, a study which treated induced pluripotent stem cell derived cardiomyocytes (iPSC-CM) with hypoxia, to mimic ischemic cardiac injury post-MI, found increased levels of both CD80 and CD86 gene levels (Screever et al., 2020). This was further strengthened in post-MI border zones of mice, in which CD80/86 expression significantly increased on both gene and protein levels. This murine model recapitulated what is seen in patients, namely, that treatment with abatacept ameliorated anti-CTLA-4 cardiac injury and resulted in better survival compared.

Additionally, intracellular dendritic cell CD80/86 signaling upon binding to CTLA-4 induces Indeolamine 2,3-dioxygenase (IDO) expression (Grohmann et al., 2002). IDO upregulation induces blockade of T-cell cycle progression, leading to reduced T-cell activation and increased T-cell apoptosis (Lohr et al., 2003; Orabona et al., 2004). Consequently, anti-CTLA-4 antibodies probably lead to reduced CD80/86 signaling in DCs, thus decreasing self-tolerance and increasing autoimmunity. This raises the possibility that pre-existing autoimmunity could be provoked by ICI therapy.

### Lymphocyte-activation gene 3 in atherosclerosis

Atherosclerosis or coronary heart disease (CAD) cases have not been reported for anti-LAG-3 therapy in clinical trials. Since anti-LAG-3 therapy was only recently approved by the FDA, large scale studies with long follow-up times are still lacking, so definitive conclusions or suggestions on whether anti-LAG-3 therapy is associated with atherosclerotic disease have yet to be made. LAG-3 was, however, found to be independently, positively correlated with CAD and present on exhausted T-cells in atherosclerotic plaques (Wherry and Kurachi, 2015; Golden et al., 2016).

In mice, single LAG-3 knockout did not lead to development of disease, but it was demonstrated that inhibition or deficiency of Gal-3 activated CD8 T-cells specifically in the tumor microenvironment, suggesting an anti-tumor effect much like PD-L1 inhibition (Kouo et al., 2015). Also similar to PD-L1, LAG-3-MHCII binding protects against Fas-mediated and drug-induced apoptosis by upregulating both MAPK/Erk and PI3K/Akt/mTOR pathways (Hemon et al., 2011). LAG-3 and PD-L1 thus might have similar functions in endothelial cells, protecting against endothelial cell death and thereby enhancing plaque stability.

### Autoimmunity in atherosclerosis

An additional mechanism of ICI-induced atherosclerosis could be through self-antigens. Several potential self-antigens were described in CAD patients, including Keratin 8 (Mihailovic et al., 2019a). Keratin eight did not increase PD-1 levels on CAD human peripheral blood mononuclear cells (PBMCs), while it did increase PD-1 mRNA levels in PBMCs obtained from controls. This shows that PD-1 can be induced by self-antigens to restrict the T-cell response, and that this mechanism is aberrant in CAD patients (Mihailovic et al., 2019b; Veluswamy et al., 2020). Additionally, gastric adenocarcinoma cells with high PD-L1 expression increase the uptake of lipids by increasing intracellular fatty acid binding protein (Fabp4/5) levels. Blockade of PD-L1 decreased Fabp4/5 expression, thereby also decreasing lipid uptake (Lin et al., 2020). Speculating, a similar process in PD-L1 expressing cells could cause reduced cellular lipid uptake upon anti-PD-L1 therapy, thus elevating free fatty acid levels at the plaque site, possibly worsening atherosclerosis. Another interesting self-antigen is apoliprotein B (apoB), the core protein of LDL cholesterol. A retrospective analysis in preprint found that lipoproteins high in apoB were risk factors for poor ICI response, especially in patients with ≥25 kg/m^2^ BMI receiving combination therapy with chemotherapy (Hu et al., 2022). While the interplay between apoB and IC remains to be elucidated, it is suggested that apoB-specific CD4 T cells can drive autoimmunity in atherosclerosis (Marchini et al., 2021). 

## Cardiac immune checkpoints

### Myocarditis

An observational retrospective study which evaluated association between ICI and cardiovascular events in a database of over 30.000 people, found that death occurred in 50% of severe myocarditis cases (Salem et al., 2018). Additionally, in the meta-analysis mentioned in the introduction, which found cardiovascular irAE to be one of the least occurring kinds but with a staggering 25% death rate, myocarditis had the highest fatality rate of 40%—at least 15% higher than all the other observed irAE in that review (Wang et al., 2018). The fatal nature lies within the fact that ICI-mediated myocarditis can be fulminant and result in cardiogenic shock and/or life-threatening ventricular arrhythmias and complete heart block. A major contributing factor in ICI-related myocarditis risk is the treatment regimen. Inhibition of the PD-1/PD-L1 pathway resulted in ICI-related myocarditis more often than inhibition of the CTLA-4 pathway (0.41 vs. 0.07%, respectively) (Salem et al., 2018). There are discrepancies in literature about this as one study in 2018 found anti-CTLA-4 to induce more myocarditis whereas a meta-analysis in 2021 again found more myocarditis cases due to anti-PD-1 (69.4%) than CTLA-4 (20%) (Mahmood et al., 2018; Rubio-Infante et al., 2021). The one clear-cut recurrent finding is that combination therapy leads to more ICI-mediated myocarditis than monotherapy. In the observational study mentioned above, combination therapy of anti-CTLA-4 with anti-PD-1 or anti-PD-L1 was indeed more common than monotherapy (1.33%) and another pharmacovigilance study suggested a 4.74-fold risk with anti-CTLA-1/PD-1 combination compared to anti-PD-1 alone (Johnson et al., 2016). Similarly, combined anti-PD-1/anti-LAG-3 therapy in recent trials reported somewhat higher numbers of myocarditis as compared to single anti-PD-1 therapy (1.7 vs. 0.6%, respectively) (Tawbi et al., 2022). With regards to other treatment modalities, there are several case-reports and studies describing cardiotoxicity, especially myocarditis, induced by combination or adjuvant therapy of ICI and radiotherapy or chemotherapy (Semper et al., 2016; Chang et al., 2018; Banfill et al., 2021; Liang et al., 2021). Therefore, research into the different means of myocardial damage inflicted by the method of treatment and the combined effect of this in the case of combination therapy is important to further optimize prevention or treatment of cardiotoxicity.

### Programmed cell death protein 1 in immune checkpoint inhibitors-mediated myocarditis

Pathology reports of patients with ICI-related myocarditis show an imbalance in immune tolerance and autoimmunity (Xavier et al., 2022). A report of two lethal cases of myocarditis in melanoma patients receiving combination anti-CTLA-4/anti-PD-1 therapy showed T-cell infiltrates in the myocardium and skeletal muscle (Johnson et al., 2016). This effect is supported by a primate study which demonstrated increased migration and activation of T-cells, and increased phagocytosis and antigen presentation in the heart after receiving PD-1, PD-L1 or CTLA-4 ICI (Ji et al., 2019). More strikingly, in the same case study, injured cardiomyocytes expressed PD-L1, indicating that direct interaction between ICI and cardiomyocytes to promote self-tolerance is possible, at least in cases of injury, and disruption by ICI therapy in this situation might be detrimental (Figure 1B) (Johnson et al., 2016). This is supported by an *in vitro* study which found that PD-L1 expression on cardiomyocytes suppressed T-cell function in mice with cardiomyopathy through downregulation of pro-inflammatory cytokines such as IFNγ (Tay et al., 2020). Though the exact mechanism behind ICI-mediated myocarditis is not understood, research has elucidated certain aspects.

Expression of IC on the endothelial cell surface and cardiomyocytes suggests a role in the protection against myocarditis and cardiac injury. In mice with CD8 T-cell-mediated myocarditis, PD-L1/2 upregulation was found on endothelial cells (Grabie et al., 2019). The expression was regulated by IFNγ and blocking IFNγ worsened the disease. Subsequent PD-L1/2 knockout or blocking therapy resulted in lethal myocarditis. Another study showed PD-L1/2 expression on murine endothelial cells injured by myocarditis. However, neither PD-L1 nor PD-L2 was detected on control mice endothelial cells (Rodig et al., 2003). This indicates a protective mechanism against cardiac injury by downregulating immune activity *via* the PD-1 pathway (Figure 1B). In addition, similarly to what is seen in PD-L1 expressing cancer cells, lymphatic endothelial cells and MHC-II expressing melanoma, intracellular PD-L1 and MHC-II signaling might inhibit apoptosis in cardiac endothelial cells and cardiomyocytes *via* MAPK/Erk and PI3K/Akt pathways (Azuma et al., 2008; Hemon et al., 2011; Gato-Cañas et al., 2017).

### Cytotoxic T-lymphocyte antigen 4 in immune checkpoint inhibitors-mediated myocarditis

A murine model by Wei at al. recapitulated ICI-mediated myocarditis and demonstrated a functional interaction between CTLA-1 and PD-1 (Wei et al., 2021). Mice with *CTLA4* haploinsufficiency alone developed myocarditis, however of the mice with complete *Pdcd1* knockout, approximately 50% of died within 3 months of age. *CTLA4* haploinsufficient mice treated with abatacept showed significant reduction in mortality and reduced myocardial immune infiltrates early in the disease.

Anti-CTLA-4-mediated myocarditis has been associated with giant cell myocarditis (Rikhi et al., 2021). In this CD4 T-cell predominant disease, chemokines such as C-X-C Motif Chemokine Receptor 3 (CXCR3) play an important role. CXCR2 is involved in several pathways, including MAP kinases and PI3K/Akt, which in turn facilitate activation, differentiation, and recruitment of CD4 T-cells. CXCR3 appears to favor recruitment of CD4 T-cells compared to CD8 T-cells, which are more associated with anti-PD-1 ICI. Additionally, increased expression of CXCR3 and its chemokine ligands have been found in giant cell myocarditis from CTLA-4 inhibition.

### Lymphocyte-activation gene 3 in immune checkpoint inhibitors-mediated myocarditis

As LAG-3 is only recently FDA approved, not many cases on ICI-mediated myocarditis have been reported yet. In a clinical trial for relatlimab-nivolumab combination therapy, myocarditis occurred in 1.7% of the combination group compared to 0.6% in the nivolumab monotherapy group. With regards to LAG-3 in animal experiments, as previously reported, knockout in mice did not lead to development of disease (Kouo et al., 2015). However, knockout of both LAG-3 and PD-1 led to the development of lethal myocarditis with T-cell infiltration and increased TNFα secretion but sustained repressive T_reg_ function, emulating trends from human clinical trials (Okazaki et al., 2011).

### Autoimmunity-induced T-cell infiltration

Recognition of shared antigens between the heart and the tumor, such endothelial cells and cardiomyocytes expressing PD-L1, or pre-existing immunity inducing an autoimmunity reaction are mechanisms proposed to induce myocardial T-cell infiltration. The latter is supported by the finding of elevated anti-troponin T antibodies in a patient presenting with ICI induced myocarditis after anti-PD-1 therapy (Martinez-Calle et al., 2018). A case of a patient developing rhabdomyolysis polymyositis after combination therapy with anti-PD-1 and anti-CTLA-4, showed elevated levels of anti-striated muscle antibodies (Bilen et al., 2016). Another candidate for autoimmunity is alpha-myosin, a cardiac specific protein. A study in preprint on the pathogenesis of ICI-mediated myocarditis found that highly clonal TCRs from three independent murine cardiac TCR repertoires were able to recognize alpha-myosin epitopes (Balko et al., 2022). Concordantly, alpha-myosin expanded T cells from the peripheral blood of two ICI-mediated myocarditis patients shared TCR clonotypes with diseased heart muscle, suggesting alpha-myosin to potentially be a clinically important autoantigen in ICI-mediated myocarditis.

### Immune checkpoint inhibitorsImmune checkpoint inhibitors-mediated cardiomyopathy

In BALB/c mice, PD-1 knockout resulted in dilated cardiomyopathy (DCM) with impaired contractile function and premature mortality (Nishimura et al., 2001). The affected heart showed immunoglobulin G (IgG) deposition on the surface of cardiomyocytes and the mice had high levels of circulating IgG autoantibodies against cardiac troponin I, with no signs of infiltrating immune cells in the myocardium, indicating an inflammatory basis for the cardiomyopathy as well as cardiomyopathy through an altered electrophysiological property of cardiomyocytes (Nishimura et al., 2001; Okazaki et al., 2003). This suggests that PD-1/PD-L1 binding aids in reducing cardiac injury (Figure 1C).

In dilated cardiomyopathy and myocardial infarction patients, PD-L1 expression was found in the myocardium and intercalated discs. In addition, they found a correlation between PD-L1 expression and left ventricular size and function (Kushnareva et al., 2022). The upregulation of PD-L1 in the myocardium points towards a role in reducing cardiac injury for PD-L1, either by downregulating the immune system through inhibition of T-cell activity *via* PD-1 or *via* inhibition of apoptosis as proposed earlier. As previously mentioned, an *in vitro* study indicated that PD-L1 expression on injured cardiomyocytes from mice with ICI-mediated DCM suppressed T-cell function (Tay et al., 2020). The proposed mechanism is that PD-1/PD-L1 interaction downregulates secretion of pro-inflammatory cytokines such as TNFγ and TNFα (Figure 1C).

A study comparing a healthy and a DCM cohort genotyped the promotor and all four exons of the *CTLA* gene to assess whether single-nucleotide-polymorphisms (SNPs) within it were associated with the diagnosis and disease course of DCM. They found a SNP in one of the exons that was significantly more frequent in the DCM patients, as well is in an additional DCM cohort they added for validation. While this *CTLA4* SNP suggests an involvement of enhanced autoimmunity in DCM (Figure 1C), their follow-up of disease course was 1-year post-diagnosis, in which it did not appear to have an influence.

## Possible therapeutic targets in downstream pathways

**FIGURE 2:**
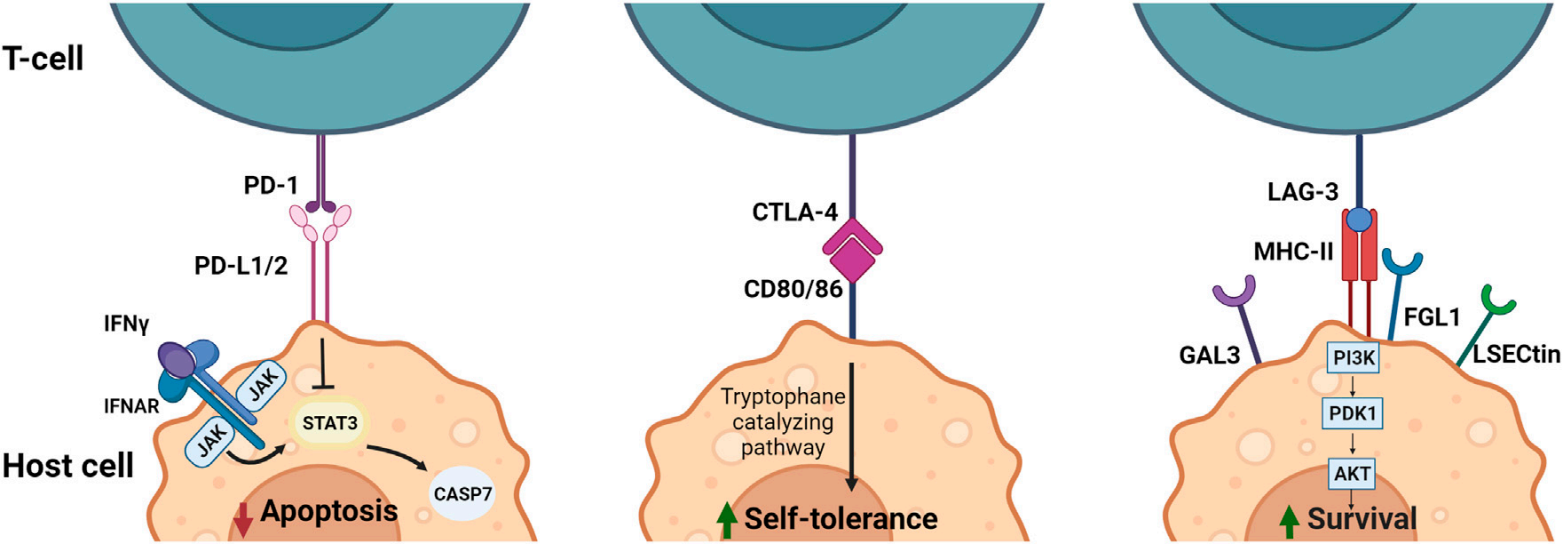
Downstream signaling and effect of immune checkpoint ligands PD-L1/2, CD80/86 and MHC-II in APCs. PD-1/PD-L1/2 interaction results in inhibition of apoptosis by interference with the IFN induced STAT3/CASP7 pathway. Blocking of this pathway by anti-PD-1 or anti-PD-L1/2 leads to less inhibition of the STAT3/CASP7 pathway and therefore increased apoptotic signaling in the presence of IFN signaling. CTLA-4/CD80/86 binding increases self-tolerance by inducing tryptophane catalyzation. Blocking of CTLA-4 leads to reduced tryptophane catalyzation and reduced self-tolerance. MHC-II/LAG-3 binding results in activation of the PI3K/Akt pathway leading to an increase in survival signals. Therefore, blocking LAG-3 results in less PI3K/Akt activation and a reduction in survival signals. CTLA-4, Cytotoxic T-Lymphocyte-Associated Protein four; FGL1, fibrinogen-like protein one; Gal3, Galectin three; IFN, Interferon; LSECtin, liver sinusoidal endothelial cell lectin; LAG-3, Lymphocyte Activation Gene-3; MHC-II, major histocompatibility complex II; PD-1, programmed cell death-1; PD-L1/2, programmed cell death-ligand 1/2. Created with BioRender.com.

Currently, immunosuppressives are the recommended first-line therapy treatment for patients with cardiovascular irAE (Andres and Ramalingam, 2019; Berg et al., 2022). However, considering varying responses to this treatment, the rapid development and approval of ICI with potent anti-tumor effects, and the fatal consequences cardiovascular irAE can illicit, exploring alternative methods of treatment applicable to more patients is necessary in order to prevent more harm. One additional reason of note for finding alternatives is prompted by the few cases in which patients treated with immunosuppressive therapies, while recovering from irAE, presented with tumor progression (Matzen et al., 2021). Downstream pathways of PD-1, PD-L1, CTLA-4, CD80/86, LAG-3 and MHCII (Figure 2 and Table 1) provide potential therapeutic targets for this as well as for immunotherapy.

**TABLE 1:** Outcomes and potential therapeutic targets based on immune checkpoints data. From left to right: current ICI targets, corresponding CVD, literature models, cardiovascular outcomes and potential therapeutic targets. CTLA-4, Cytotoxic T-Lymphocyte-Associated Protein four; FGL1, fibrinogen-like protein one; Gal3, Galectin three; IFNy, Interferon gamma; LSECtin, liver sinusoidal endothelial cell lectin; LAG-3, Lymphocyte Activation Gene-3; MHC-II, major histocompatibility complex II; PD-1, programmed cell death-1; PD-L1/2, programmed cell death-ligand 1/2.

IC/IC ligand	CVD	Model	Outcome	Possible target
CTLA-4	Atherosclerosis	PD-1/PD-L1 KO: Increased plaque size; higher T-cell numbers; increased TNFα; increased T-cell activation; enhanced cytotoxic T-cell activity (Gotsman et al., 2007; Bu et al., 2011)	-1.5-3-fold increase in aortic lesion areas	TNFα
			-2-fold increase in lesion of aortic arch	
		High PD-1 expression on T-cells in the atherosclerotic plaque (Fernandez et al., 2019)	-Significantly more PD-1 on CD8 T-cells in plaque than blood	
		*Pdl1* ^−/−^ increased lymphatic endothelial cell apoptosis (Lucas et al., 2018)	-±20% more caspases	Caspases
		PD-L1 block: no/reduced upregulation of surface IC and cytokine production in T_reg_ (Chen et al., 2016; Mazanet and Hughes, 2022)	-26.43% less PD-1	T_reg,_ IL-10 and TGF-βIFNγ
			-15.63% less CTLA-4	
			-3.8-fold decrease in IL-10	
			-2-fold decrease in TGF-β1	
		Endothelial PD-L1/2 block: enhanced IFNγ secretion by CD8 T-cells (Rodig et al., 2003)	-±35% more IFNγ with PD-L1 block	IFNγ
			-±45% more IFNγ with PD-L2 block	
	Myocarditis	*PD-L1/2* ^−/−^CMy-mOva mice: lethal myocarditis; PD-L1 upregulation is IFNγ dependent (Grabie et al., 2007)	-Mortality down by 50% at day 10	
	Atherosclerosis and Myocarditis	PD-L1 signaling: inhibition of IFNγ-induced apoptosis in cancer cells through STAT3/Casp7 (Azuma et al., 2008; Gato-Cañas et al., 2017)	-Silencing of STAT3 and CASP7 abrogated IFNβ lethality	STAT3/Casp7
	Cardiomyopathy	BALB/c–PD-1 KO mice: DCM with impaired contractile function; IgG deposition on cardiomyocytes; high levels of circulating anti-troponin IgG (Nishimura et al., 2001; Okazaki et al., 2003)	-Premature death in PD-1^−/−^ at as early as 5 weeks	
			-57% decrease in ventricular fractional shortening	
			-28.5% reduction of ejection fraction	
		DCM: associated with increased and widespread cardiac PD-L1 expression (Kushnareva et al., 2022)	-PD-L1 on endothelial cells and membrane surface	
	Atherosclerosis	Anti-CTLA-4 in *Ldlr* ^*−/−*^ mice: increased plaque size (Poels et al., 2020)	-Doubled plaque area (0.8–3.2 mm^2^)	
			-±5% increase in necrotic core	
		CTLA-4-Tg/*Apoe* ^−/−^ mice: Reduced plaque formation; reduced CD4 T-cell numbers; reduced T-cell proliferation; reduced proinflammatory cytokine production (Matsumoto et al., 2016)	-35% decrease in lesion size in males mice	
			-26% decrease in lesion size in female mice	
	Cardiomyopathy	*CLTA4* gene variant led to increased risk of DCM (Ruppert et al., 2010)	-*CTLA4* SNP 7.4% more frequent in DCM patients	
CD80/86	Myocarditis	Increased self- tolerance by inducing tryptophan catabolism (Grohmann et al., 2002)	-Long-term islet engraftment in mice when CTLA-4 activation was blocked	Tryptophan, IDO, IFNγ
CTLA-4 and PD-1/PD-L1	Myocarditis	Myocarditis in ICI treated patients: increased with combination therapy (Johnson et al., 2016)	-0.27% chance of myocarditis with combination ICI vs. 0.07 with anti-PD-1
		Ctla4^+/−^ Pdcd1^−/−^ mice: myocarditis with cardiac T-cell infiltration; reduced T_reg_ counts	-50% mortality by 3 months
		Abatacept treatment reduced mortality (Wei et al., 2021)	-Abatacept increased survival
LAG-3	Atherosclerosis, hypertension, myocarditis, and cardiomyopathy	LAG-3 KO mice: no disease onset	-Maximum IFNγ levels in LAG-3^−/−^ mice
		Gal-3 depletion: increased pro-inflammatory immune cells (Kouo et al., 2015)	-Significantly more T-cells and dendritic cells upon Gal-3 depletion
LAG-3 and PD-1	Myocarditis	BALB/c *Pdcd1−/−Lag3* ^*aida/aida*^ mice: lethal myocarditis with T-cell infiltration; increased TNFα secretion; sustained T_reg_ function (Okazaki et al., 2011)	-Premature death at 5 weeks	TNFα
				T_reg_
MHCII	Atherosclerosis and myocarditis	LAG-3-MHCII:promotes survival in melanoma by upregulation of MAPK/Erk and PI3K/Akt pathways (Hemon et al., 2011)	-24% less cell death at 24 h	MAPK/Erk
				PI3K/Akt/mTOR

The JAK/STAT pathway is involved in both PD-L1/2 and CD80/86 signaling, promoting transcription of multiple cytokines seemingly involved in irAE (Kubo et al., 2014; Doi et al., 2017). Multiple JAK inhibitors have been approved for application in autoimmune disorders and can therefore potentially also be used for treatment of cardiovascular irAE. Two successful cases with PAN-JAK-inhibitor (tofacitinib) treatment for ICI-mediated myocarditis in cancer patients are described by Liu et al. (Liu and Jiang, 2020). Both patients recovered from myocarditis rapidly, with no signs of adverse effects. In a retrospective study comparing ICI-mediated myocarditis patients responsive or resistant to corticosteroid treatment, 11 resistant patients were treated with tofacitinib of which 7 recovered with no adverse effects (Wang et al., 2021). Notably, JAK/STAT3 has shown to be pro-oncogenic and subsequently inhibition may result in a synergistic anti-tumor effect with ICI (Berg et al., 2022). However, treatment with JAK/STAT inhibitors requires caution with regards to pro-tumor effects. Since STAT1 is known to be important in the antitumor immune reaction through induction of IFNγ secretion, inhibition of STAT1 *via* JAK1 or JAK2 may result in reduced anti-tumor clearance. Therefore, specific inhibition of JAK/STAT inhibition might be beneficial to treat irAE.

Alternatively, the MAPK/Erk and PI3K/Akt/mTOR pathways are behind proliferation and inflammation (Table 1). One of the targets that is currently investigated is mTOR. Combined anti-PD-1 therapy and mTOR inhibition in a melanoma patient with ICI-induced allograft rejection resulted in retained anti-PD-1 tumor efficacy, while promoting tolerance to the allograft (Esfahani et al., 2019). IFNγ-producing CD4 and CD8 T-cells persisted in circulation, while proinflammatory cytokines IL-6, TNFα and IL-17A returned to baseline levels. This suggests a possible new target for reduction of anti-PD-1 induced toxicity, while maintaining anti-tumor efficacy. MNK1/2 is a factor activated downstream of both MAPK/Erk and PI3K/Akt/mTOR pathways and facilitates transcription of mRNAs that promote cell proliferation and survival. Inhibition of MNK1/2 has demonstrated reduced oncogenicity and metastasis in melanoma patients (Zhan et al., 2017). Contrarily, MNK1/2 is also involved in production of proinflammatory cytokines IL-6 and TNFα (Joshi and Platanias, 2014).

Finally, the tryptophane catalyzing pathway induced by CD80/86 signaling in DCs is associated with reduced T-cell activity and increased self-tolerance (Fallarino et al., 2003). An important molecule in the tryptophan catalyzation is indoleamine 2,3-dioxygenase 1 (IDO1). Therefore, this may be an additional downstream target for cancer immunotherapy, as IDO has also been reported to be overexpressed in some tumor types causing immune evasion. Multiple clinical trials targeting IDO as cancer therapy are ongoing, including inhibitors and a vaccine (Tang et al., 2021). Additionally, a clinical phase I/II with an IDO inhibitor (BMS-986205) in combination with nivolumab showed promising results, with response rates of 46% in bladder cancer and 25% in cervical cancer (Blocking IDO1 Helps Shrink Bladder, Cervical Tumors, 2018). Subsequently, a phase III trial was started, in addition to clinical trials to study the IDO inhibitor in combination with ipilimumab and relatlimab (Long et al., 2019; Tang et al., 2021). However, the failure of a phase III trial looking into pembrolizumab (anti-PD-1) in combination with anti-IDO put a hold to the development of IDO1 inhibitors and demanded more insight in the mechanism behind IDO1 inhibitors.

## Perspective

PD-1, PD-L1, CTLA-4, and LAG-3 are currently approved therapeutical targets in cancer therapy. However, in nearly 10% of patients treated with ICI potentially life-threatening cardiovascular irAE occur (Xavier et al., 2022). And with the growing number of ICI being approved and used in the clinics, the incidence of cardiovascular irAE is expected to increase. IC downregulate the immune system by modulating several pathways that are involved in T-cell activation, differentiation, and survival, thereby reducing inflammation, and promoting tolerance. Additionally, the IC ligands have shown to have additional intracellular signaling mechanisms to reduce apoptosis and promote self-tolerance in target cells like endothelial cells and cardiomyocytes. The exact mechanisms behind the three ICI-mediated diseases, atherosclerosis, myocarditis and cardiomyopathy, are not completely understood and future studies are warranted. Currently, irAE management with corticosteroids is not beneficial for everyone and downstream targets are urgently needed. Cases with successful treatment for ICI-mediated myocarditis, e.g. Abatacept (NCT05335928), have been reported with and randomized trials are underway. Additionally, more research focusing on downstream pathways of IC ligand host-cells, such as the JAK/STAT, MAP/Erk/mTOR, PI3K/Akt pathways and IDO1, as possible therapeutic targets is required.

While understanding the role of IC in the CV system is important, surveillance and prevention play an equally important role in the clinical setting. Serial monitoring with echocardiography (ECG), electrocardiogram and biomarkers (i.e. troponin) is advised for immunotherapy, chemotherapy and radiotherapy (Banfill et al., 2021; Stein-Merlob et al., 2021; Huang et al., 2022). According to the latest guidelines of the European Society of Cardiology, all ICI-treated patients should have ECG and troponin measured at baseline, with additional ECG monitoring in patients with elevated baseline troponin levels (Lyon et al., 2022). In cases of newly developed ECG abnormalities, biomarker changes or cardiac symptoms at any time during the course of ICI treatment, the guidelines recommend immediate cardio-oncology evaluation with additional ECG for left ventricular ejection fraction and strain analysis. Cardiac magnetic resonance is recommended when myocarditis is suspected.

## Conclusion

ICI therapies have provided for successful therapeutic options and improved prognosis for a large group of cancer patients, however their interaction with the cardiovascular system can be detrimental. Inhibition of IC ligand interactions have been indicated to accelerate onset and development of atherosclerosis, increase inflammation and myocardial infiltration which causes fatal myocarditis, and it is proposed that it induces autoimmunity and electrophysiological alterations of cardiomyocytes leading to cardiomyopathy. Insights into the mechanisms behind IC and cardiovascular irAE are important to investigate in order to: 1) determine preexisting risk factors for better patient selection, 2) unravel treatment possibilities for cardiovascular irAE with, ideally, sustained anti-tumor ICI efficacy, and 3) indicate possible new targets for cancer therapy.
